# Improved efficiency of butanol production by absorbent fermentation with a renewable carrier

**DOI:** 10.1186/1754-6834-6-121

**Published:** 2013-08-26

**Authors:** Qin He, HongZhang Chen

**Affiliations:** 1 National Key Laboratory of Biochemical Engineering, Institute of Process Engineering, Chinese Academy of Sciences, Beijing 100190, People’s Republic of China

**Keywords:** Absorbent fermentation, Butanol, Steam explosion, Porous medium

## Abstract

**Background:**

Biobutanol production is still not economically competitive because of some principal drawbacks including high cost in feedstock consumption, low butanol concentration in the fermentation broth caused by severe product inhibition. An alternative fermentation mode is becoming an urgent requirement to solve these problems. Biobutanol production by absorbent fermentation with a renewable carrier, i.e. pretreated straw materials, is studied in this paper.

**Results:**

Compared with other types of porous media, alkali-treated steam-exploded straw was proved to be a suitable carrier for absorbent fermentation of butanol. The Acetone-Butanol-Ethanol (ABE) concentration increased by 52% compared with submerged culture at an initial glucose concentration of 65 g/L. The adsorption of ABE solvent on substrate and increased bacterial concentration alleviated the end product inhibition and partly explained this positive effect. The steam pretreatment conditions, solid–liquid ratio, substrate types and substrate concentration were also investigated. Steam-explosion at 1.1 MPa for 4 min and solid–liquid ratio of 1:10 was shown to be the optimum. Glucose showed a great advantage over xylose, and higher glucose content was more conducive to biobutanol production. However, the yield of solvent decreased with the increased initial sugar concentration. Considering comprehensively, 100 g/L initial glucose was considered to be the optimum.

**Conclusions:**

This work demonstrated an effective approach of improved butanol fermentation and its probable mechanisms of this positive effect, i.e. the adsorption of ABE solvent and the adhesion of bacteria on porous substrate accounted for the production improvement and the proportional variation of solvent constituents.

## Background

Butanol is expected to play a major role in next-generation of biofuels, considering its characteristics of higher miscibility with gasoline and energy density than ethanol. Nowadays biological butanol production has gained renewed interest due to the anxieties over global oil supply and its impact on social and economic development now. However, because of its principal problems: high cost in feedstock consumption, low butanol concentration in the fermentation broth caused by severe product inhibition, biobutanol production is still not economically competitive [1].

Solid-state fermentation (SSF), which involves the growth of microorganisms on moist solid substrate in the absence of free water, has considerable economical potential in producing products for the food, feed, pharmaceutical and agricultural industries [2]. However, owing to the great success of large-scale submerged fermentation (SmF), SSF has been almost neglected for several decades except in some food fermentation and the production of enzymes such as cellulases, etc. Fortunately, over the last decade, SSF has attracted renewed attention because of its certain advantages over SmF [3,4], including improved product characteristics, higher product yields, easier product recovery and reduced energy requirements.

However, in SSF, the solid medium would degrade during the fermentation, resulting in the changes of physical features with the media, and consequently the capacity of heat and mass transfer in the medium could be reduced. For example, oats would shrink owing to the utilization of starch and the evaporation of water, and this would lead to reduced abilities of mass and heat transfer [5]. The disadvantages can be overcome via using an inert support with a more constant physical structure throughout the process, i.e. the absorbent fermentation (AF). It’s a second type of SSF which enables improved control of heat and mass transfer. Additionally, compared to traditional SSF, AF presents more advantages such as easier preparation of medium and more convenient product recovery [2].

In AF, a special kind of SSF, the solid support can be of natural origin that serves only as carrier for microorganisms such as hemp, perlite, sugarcane bagasse, and vermiculite [2,5-7] or artificial synthetic compounds such as polyurethane foam (PUF) [8]. Many products, such as polysaccharides [9], enzymes [10], and organic acids [11], have been produced by AF, but there are no related reports on butanol fermentation.

In this paper, in order to select a suitable carrier for absorbent fermentation of Acetone-Butanol-Ethanol (ABE), various types of porous media were used. The adsorption of product on substrate was stressed to elucidate the properties of this butanol fermentation process. In addition, the effects of some important parameters in AF were investigated.

## Results and Discussion

### Choice of absorbent for butanol fermentation

**Figure 1:**
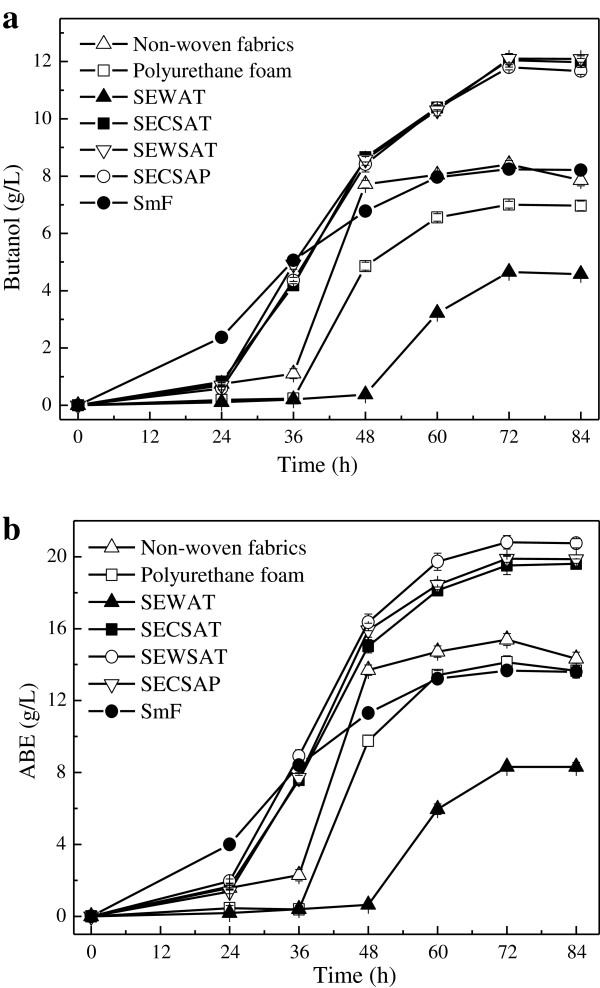
**Product concentration in different absorbent cultures with the same initial glucose concentration of 65 g/L. (a)** Butanol concentration; **(b)** ABE concentration.

An inert substrate can be used as a support for microbial growth after impregnation with the appropriate nutrient solution. A high absorption capacity of cells is a critical requirement of the solid support to be used [12]. Different substrates were selected for butanol fermentation, including non-woven fabric, polyurethane foam, and lignocellulosic substrates, such as alkali-treated steam-exploded corn stover (SECSAT), alkali-treated steam-exploded wheat straw (SEWSAT), alkali-treated steam-exploded wood (SEWAT) and alkaline peroxide treated steam-exploded corn stover (SECSAP). The results of absorbent fermentation were shown in Figure 1. In submerged fermentation as the control, 8.24 g/L butanol and 13.67 g/L ABE solvent were obtained at an initial glucose concentration of 65 g/L at 72 h. In absorbent fermentation, increased concentrations of butanol and ABE could be achieved when SECSAT, SEWSAT and SECSAP were used as the absorbents, and the highest concentrations reached 12.10 g/L and 20.80 g/L in SEWSAT model, respectively (We employed corn stover rather than wheat straw as the material in the following studies, mainly because of the higher biomass per acre). The ABE concentration increased by 52% compared with that in SmF.

Batch fermentation for butanol production offers easy operation and reduced risk of contamination [13], and much research effort has been exerted to improve the batch fermentation efficiency by applying varied fermen-tation strategies. Tran et al. reported improvement in butanol production from starch by co-culture of an amylase producing strain with *Clostridium butylicum* TISTR 1032, and the production was improved by 5.4 times as ABE obtained by soluble starch [14]. Presently, lots of reports on batch fermentation technology were focused on the simultaneous fermentation and separation, e.g. *in situ* gas-stripping, this technology could enhance the ABE concentration by 21% [15].

On the other hand, non-woven fabric did not offer obvious enhancement on butanol production of 8.41 g/L, whereas polyurethane foam provided less butanol production of 7.01 g/L, and when it came to the SEWAT model, the butanol concentration was only 4.65 g/L. Specially speaking, the phenomenon in polyurethane foam test was greatly different from former reports on AF [9-11].

Two main reasons causing the fermentation differences were proposed. First, the difference in ABE solvent adsorption affinities of the substrate was the probable driving factor. As described in a former report, butanol adsorption by carrier was driven by hydrophobic interactions [16]. Improved biocompatibility between carrier and product would enhance the fermentation, and the suggesting carrier included poly(styrene-co-divinylbenzene) materials which possessed the greatest n-butanol affinity [16]. Thus, polyurethane foam showed improper for butanol absorption because its varied adsorption affinities toward ABE solvent and other products. Second, different materials resulted in different increases of butanol production due to unique porosity characteristics. Similar amount of butanol increases were observed in absorbed SECSAT and SEWSAT fermentations (Table 1), which could be explained by a higher porosity and specific surface area. For example, compared with the porosity of 70%-80% of SECSAT, SECSAP and SEWSAT, SEWAT and non-woven fabric presented a less porosity. Furthermore, despite the higher porosity, the polyurethane foam test behaved decreased fermentation efficiency of butanol because of the absorption selectivity.

**Table 1:** Physical properties of absorbents

**Materials**	**Specific gravity (g/mL)**	**Bulk density (g/mL)**	**Porosity (%)**	**Specific surface area (m**^**2**^**/g)**
SECSAT	0.983±0.053	0.188±0.017	75.7±2.3	0.231±0.013
SECSAP	1.021±0.067	0.126±0.009	82.7±2.2	0.171±0.009
SEWSAT	1.169±0.043	0.149±0.013	82.9±2.2	0.138±0.003
SEWAT	0.619±0.040	0.352±0.007	35.1±1.0	0.116±0.003
Non-woven fabric	0.402±0.035	0.15±0.010	50.2±0.7	0.127±0.002
Polyurethane foam	1.509±0.058	0.105±0.008	89.7±2.4	0.217±0.005

In addition to the porosity, the pore size could be important in fermentation. For porous material, pore size distribution was discovered to play an important role in mass transfer [17], and it might affect the fermentation process largely because of its selective adsorption of appropriate sizes of products and cells [16].

**Figure 2:**
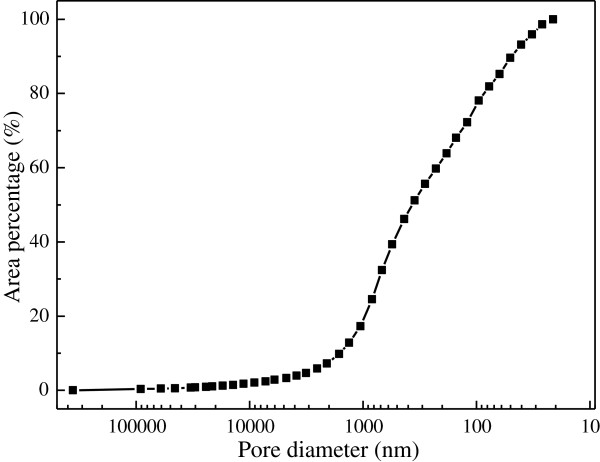
Pore size distribution of SECSAT.

Mercury porosimetry method was used for evaluation of the pore size distribution in SECSAT. The pore sizes of SECSAT ranged from mesoporous to macroporous (Figure 2), i.e. reach a few hundred nanometers or several microns. Thus, the microorganisms could be absorbed closely on the surface or inner cover of carrier easily, and could contact with fermentation broth and solid substrate simultaneously to form a two-phase partitioning system employing liquid and solid phases. The interface would be the area for heat and mass transfer. As for non-woven fabric, a kind of hydrophobic carrier, due to the smaller pore size and lower porosity, the fermentation efficiency showed little difference from that in submerged fermentation.

### Adsorption of ABE solvent and bacterial adhesion onto SECSAT

#### Adsorption of ABE solvent onto SECSAT

**Figure 3:**
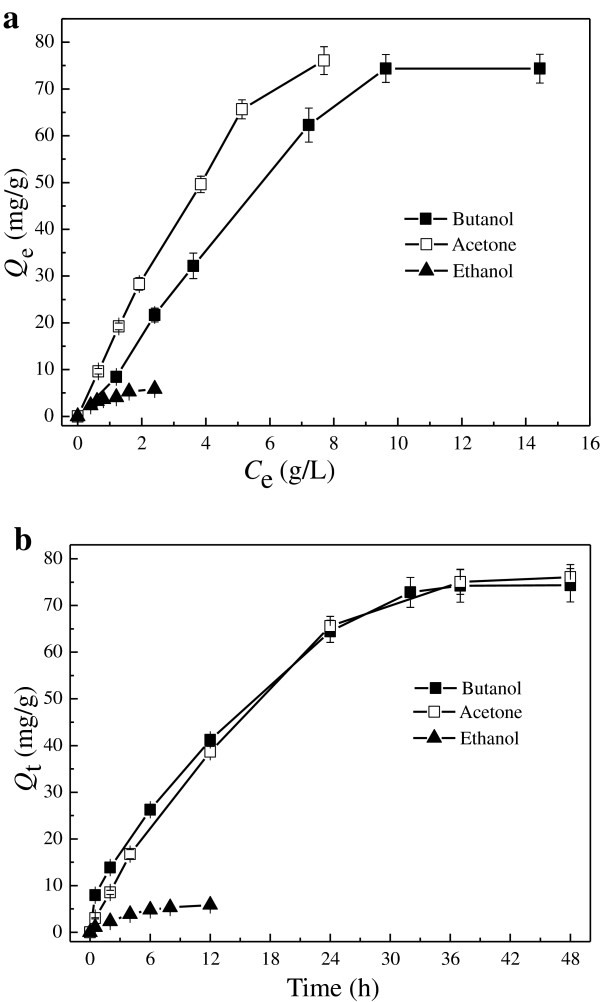
**Adsorption properties of ABE on SECSAT at 37°C. (a)** Adsorption isotherms of ABE on SECSAT at 37°C; **(b)** Adsorption kinetics of ABE on SECSAT at 37°C.

The pores with different sizes were the passages for mass transfer and the adsorption was the main form of mass transfer in ABE fermentation because of the only support role of the porous carrier in fermentation [18]. As butanol, acetone and ethanol were the main components in the products, simulated solvent of which initial concentrations of butanol, acetone and ethanol were 14.435 g/L, 7.694 g/L and 2.402 g/L respectively, was applied to study the adsorption process. The adsorption data expressed the accumulation degree of these three components onto adsorbent surface respectively (Figure 3).

Firstly, the adsorption equilibriums of acetone and butanol both reached 70 mg/g substrate or even higher, i.e. about 5 g/L acetone or butanol was adsorbed onto SECSAT in the simulated solvent (Figure 3 (a)). However, the adsorption of ethanol was lower. Taken together, acetone was preferentially adsorbed onto the carrier than butanol and ethanol. The high affinity between acetone and the absorbed carrier explained the promoted acetone production in the presence of SECSAT [18]. The adsorption of ABE alleviated product (butanol) inhibition, which could furthermore enhance the ABE production, and after fermentation the solvents could be easily separated from the carrier. This made the operation for carrier reuse easier.

The adsorption kinetics of ABE on SECSAT at 37°C was showed in Figure 3 (b). Although the concentration of butanol was twice as much as that of acetone in liquid, the adsorption speed was presented similar, while the adsorption speed and amount for ethanol were relatively lower. The priority of adsorption on vector was probably affected by two factors: (1) the hydrophobic nature of the solvent: the stronger the hydrophobicity of the solvent, the more adsorbed onto the carrier; (2) the interaction between the molecules of the same solute: weak interaction was better for adsorption onto the carrier. Although the hydrophobicity of acetone was lower than that of butanol, the interaction between the acetone molecules could be less than that between butanol molecules at the same temperature due to the lower boiling point, thus favored the adsorption of acetone [19].

#### Bacterial adhesion onto SECSAT

**Figure 4:**
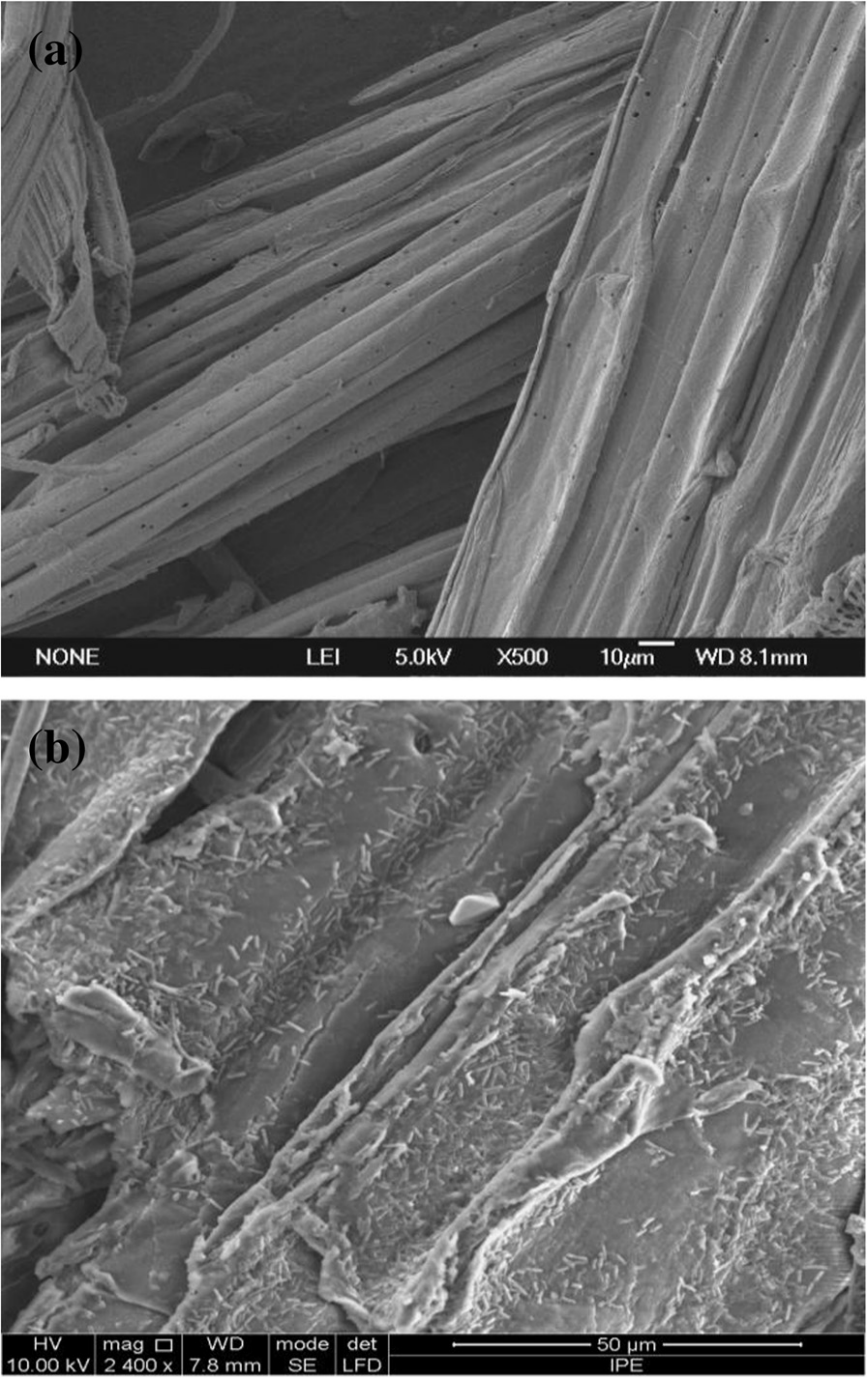
**The morphology of SECSAT before and after fermentation. (a)** Scanning electron micrograph of SECSAT; **(b)** Scanning electron micrograph showing the distribution of bacteria on SECSAT at 72 h of incubation.

The slightly larger size scale of the carrier pore than bacteria cell made the absorption of bacteria on the carrier possible, i.e. the size of *C. acetobutylicum* cell was about 0.6-0.9 *μ*m × 2.4-4.7 *μ*m, and the pore diameter of the carrier ranged from several nanometers to dozens of microns with the average value of 3.5 *μ*m. The scanning electron micrographs of SECSAT before and after fermentation were showed in Figure 4. Considerable bacteria were uniformly adhered onto SECSAT and polyurethane foam, whereas few bacteria absorbed on SEWAT or non-woven fabric (The figures were not shown). The uniform distribution of cells onto the carrier in the scanning electron micrograph was similar as a former report on the *Lactobacillus casei* absorption on polyurethane foam which indicating that polyurethane foam cubes could provide a platform for uniform distribution of the microorganisms [11].

Besides, the adsorption amount of cells reached about 0.07 g/g dry substrate, i.e. 4.67 g/L fermentation broth; meantime the amount of biomass in broth reached 3.5 g/L. The biomass amount was higher than that of 2.1g/L obtained in submerged fermentation. The enhanced biomass in broth and adsorbed bacteria was apparently another reason for butanol production improvement because high cell concentrations resulted in high reactor productivity [20].

In this sense, another reason for the enhancement of product yield was probably the effect of adhesive force of inert support on cells. When the carrier had pores that were large in relative to the cell dimensions, it was possible to find adhesion within the pores [21], and the freedom in cellular movement could be restricted in two main ways: adsorption of cells (either between the cells themselves or to a solid carrier surface) with physical and chemical bonds, or physical entrapment of cells within the carriers [22]. Thus, the absorbed cells were considered to be attached on the carrier.

Adhesion could prevent cells from being washed away when they find themselves in a nourishing environment, and allow them to form biofilms that offer protection from hazardous conditions [23]. Hence the efficiency of holding ABE solvent and uniform dispersal of the inoculums resulted in alleviated catabolite repression, and consequently higher yield of butanol was reached than the submerged fermentation. Another fermentation strategy, self-immobilized and its conception was reported by Bai [24]. They further reviewed ethanol fermentation with flocculating yeast by comparing with immobilized yeast cells with supporting materials and free yeast and achieved the industrialization with the working volume of 400 m^3^[25].

As for the technically advantages of this technology, porous lignocellulosic carrier almost behaved all the characteristics of the ideal cell supports. Particularly, it could solve problems of cell injury caused by the immobilized cells and shear stress, could improve the cell density to facilitate large-scale cell culture, and at the same time could greatly alleviate product inhibition on microorganisms. In comparison, cells immobilized with supporting materials were not suitable because of cell injury and mechanically constrained, etc., whereas free cells provided lower product yield.

### Optimization of butanol production by absorbent fermentation

#### Effect of pretreatment severity of corn stover on butanol fermentation

SECSAT samples obtained under different pretreatment severities were used for butanol fermentation. Results showed that steam-explosion pretreatment severity significantly influenced butanol production by absorbent fermentation (Table 2). At lower severities, the effects were not distinct from those in submerged fermentation. However, when it came to higher severities, the butanol titers turned out lower. The optimum pretreatment condition of steam explosion was at 1.1 MPa for 4 min with the log_10_*R* of 3.05. The change of porosity and pore size of the substrates, and the production of inhibitors contributed to the impact of steam explosion on butanol fermentation. Optimum steam-explosion severity enhanced porosity and promoted fermentation performance, while higher severity brought minute fragments with small and closed pores, and lower severity could enhance porosity and specific surface area slightly.

**Table 2:** **Effect of pretreatment severity of corn stover on butanol fermentation**^**a**^

**Log**_**10 **_**R**	**P (MPa)**	**t (min)**	**T (°C)**	**Butanol (g/L)**	**Total ABE (g/L)**
2.75	1.1	2	184.1	8.81±0.03	16.23
3.05	1.1	4	184.1	12.10±0.08	20.81
3.35	1.2	4.5	188.0	10.98±0.17	19.32
3.50	1.3	5	191.6	9.53±0.12	18.62
3.68	1.5	5	198.3	9.40±0.07	17.31
3.89	1.4	10	195.1	9.28±0.05	17.07

^a^Culture conditions: Using 65 g/L glucose as carbon source, SECSAT as the absorbed substrate, 72 h.

#### Effect of liquid–solid ratio on butanol fermentation

Liquid–solid ratio (the weight ratio of water to dry solid substrate) was an important parameter in absorbent fermentation. The effect of liquid–solid ratio on butanol fermentation under the same culture condition was studied (Table 3). The highest butanol production was achieved in the liquid–solid ratio of 1:10, and large quantities of sugars remained in the broth in test of lower liquid–solid ratio where less amount of free water restricted sufficient access of microorganisms to the substrates. On the other hand, However, higher liquid–solid ratio would increase the amount of free water and more sugars would be consumed for bacterial growth in order to reach a corresponding cell concentration, thus, the yield of ABE would be decreased.

**Table 3:** **Effect of liquid–solid ratio on butanol fermentation**^**a**^

**Liquid–solid ratio**	**Ethanol (g/L)**	**Acetone (g/L)**	**Butanol (g/L)**	**Total ABE (g/L)**
3:1	0.44±0.03	1.25±0.02	2.37±0.05	4.06
5:1	0.38±0.02	3.05±0.03	3.63±0.03	7.06
10:1	0.40±0.01	5.71±0.03	8.44±0.02	14.55
15:1	0.35±0.02	5.22±0.04	7.75±0.05	13.32
20:1	0.32±0.01	4.39±0.03	7.00±0.06	11.71

^a^Culture conditions: Using 54 g/L xylose as carbon source, SECSAT as the absorbed substrate, 72 h.

#### Effect of sugar mixtures on butanol fermentation

Hemicellulose hydrolysate has been becoming an important carbon source for butanol fermentation in terms of reducing the cost of industrial applications. To utilize the mixture sugars, i.e. both pentose and hexose, was of great significance, since they present in hydrolysates of agricultural and forestry residues. *C. acetobutylicum*, as a strict anaerobic bacterium, was one of the few microbes that are able to ferment xylose and other pentoses [26]. In this paper, glucose, xylose and the mixture of both were used as model substrates to investigate their fermentability by *C. acetobutylicum*. The fermentation efficiencies of different glucose and xylose mixtures proportionally prepared was showed in Table 4. It was evident that the sugar utilization rate, solvent yield and butanol concentration were higher when glucose was used as a sole carbon source other than xylose. When cultured in sugar mixtures, a decreased fermentability was found with an increased proportion of xylose.

**Table 4:** **ABE production and glucose/xylose utilization with SECSAT as absorbent**^**a**^

**Carbon sources**	**Total sugar (g/L)**	**Consumption rate of sugar (%)**	**Ethanol (g/L)**	**Acetone (g/L)**	**Butanol (g/L)**	**Acetic acid (g/L)**	**Butyric acid (g/L)**	**Total solvent (g/L)**	**Yield of ABE (g/g)**
Glucose	60	98.73±0.67	1.00±0.01	7.47±0.04	10.07±0.04	1.17±0.06	0.85±0.06	18.54	0.31
Xylose	60	92.88±1.17	0.42±0.02	5.93±0.05	8.76±0.04	2.91±0.16	2.01±0.13	15.11	0.27
Glucose/Xylose	40/20	97.81±0.33	0.95±0.02	7.14±0.04	9.92±0.08	2.56±0.13	2.50±0.15	18.01	0.31
Glucose/Xylose	30/30	97.38±0.09	0.92±0.02	6.97±0.03	9.73±0.04	2.48±0.19	2.55±0.23	17.62	0.30
Glucose/Xylose	20/40	95.47±0.15	0.74±0.05	6.88±0.03	9.63±0.07	1.25±0.11	2.25±0.17	17.25	0.30
Glucose/Xylose	15/45	93.79±0.22	0.67±0.04	6.47±0.15	9.46±0.07	2.79±0.17	1.18±0.12	16.60	0.29

^a^Culture conditions: Using SECSAT as the absorbed substrate, the solid–liquid ratio was 1:10, 72 h.

The results were coincident with those of a former study for the fermentability of glucose, xylose and their mixtures [27]. During the fermentation, the ABE concentration increased gradually until 60 h and thereafter remained nearly constant, probably due to the end product inhibition on the microorganisms. The final ABE concentration in mixed glucose/xylose culture was lower than the average value obtained in separate glucose and xylose cultures, indicating that glucose partially inhibited xylose utilization in glucose/xylose mixtures.

**Figure 5:**
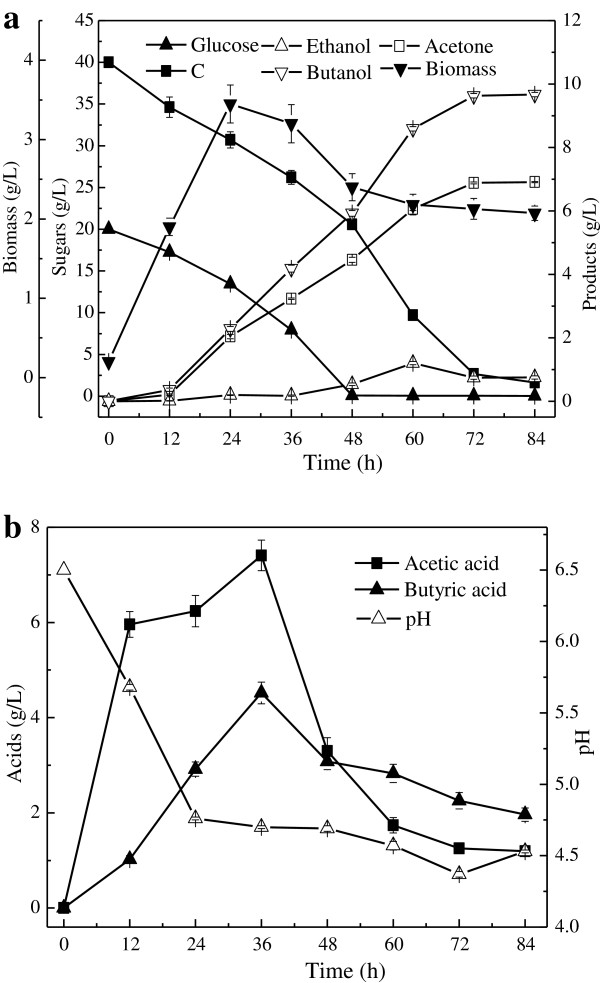
**ABE production in complex medium containing 20 g/L glucose and 40 g/L xylose by AF. (a)** Bacterial biomass, sugar consumption and solvent production;**(b)** Acids production and pH change.

The metabolic process of *C. acetobutylicum* ATCC 824 in complex medium composed of 20 g/L glucose and 40 g/L xylose was studied (Figure 5). The glucose was completely consumed at 48 h, the ABE accumulated to 17.25 g/L and its yield reached 0.31 g/g at 72 h. We know, when glucose was present, utilization of xylose was generally inhibited. In absorbent fermentation, xylose and glucose could be used simultaneously instead of that the xylose utilization was strongly inhibited in submerged fermentation. Nevertheless, there occurred a significant increase of xylose consumption rate when glucose was used up, indicating a less glucose-mediated catabolic repression remained. Additionally, the free biomass achieved maximum at 24 h, and the final pH was steady at 4.5, suitable for solvent production.

#### Effect of initial sugar concentration on butanol fermentation

Product inhibition was one of the problems for industry-level butanol fermentation. Ounine et al. [28] found that xylose permease was strongly inhibited at a butanol concentration of 8 g/L whereas glucose permease was inhibited at 12 g/L. Lee et al. [29] suggested that initial glucose concentration could not be above 90 g/L due to severe growth inhibition.

**Figure 6:**
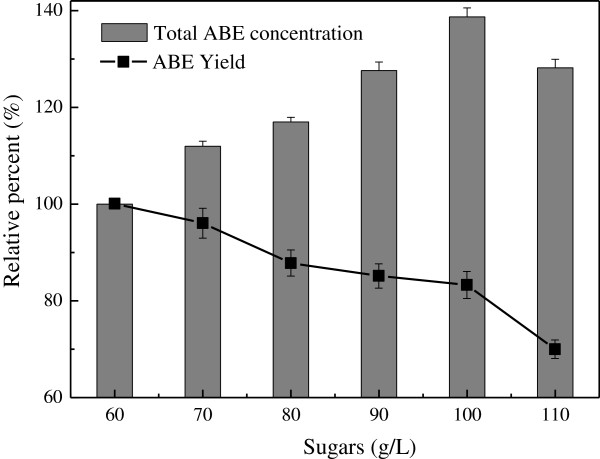
Effect of substrate concentration on butanol fermentation.

In the absorbent fermentation, the effect of substrate concentration on butanol fermentation was also investigated to determine the appropriate sugar concentration (Figure 6). Results showed that the total solvent concentration increased with increasing substrate concentration. The highest total solvent production was found at glucose concentration of 100 g/L, which was 1.39 times of that achieved at 60 g/L glucose; however the solvent yield was only 83.2% of the latter, i.e. 0.26 g/g, it thereby demonstrated from the figure that the solvent yield decreased with the increased substrate concentration. It can be explained that, absorbent fermentation only reduced the product feedback inhibition to a certain extent. Based on an overall consideration of various factors, we preferred to choose the concentration of 100 g/L glucose as appropriate.

In comparison, the fermentation efficiency was better than the report that in the pH 5.5, 69.8 g/L of glucose were catabolized and the yield of solvents on glucose was only 0.20 g/g in an initial glucose concentration of 80 g/L with the same strain used [29].

## Conclusions

Alkali-treated steam-exploded straw, a new type of lignocellulosic porous carrier, was proved to be more effective for butanol production by absorbent fermentation than other porous carriers. The adsorption of ABE solvent and the adhesion of bacteria on substrate accounted for butanol production improvement and the proportional variation of solvent constituents. The culture conditions for butanol production by absorbent fermentation were studied. Steam-explosion pretreatment at 1.1 MPa for 4 min, solid–liquid ratio of 1:10 and 100 g/L initial glucose was considered to be the optimum concerning the trade-off between production and yield. In sum, absorbent fermentation with the lignocellulosic porous carrier, an abundant, cheap and renewable substrate, increased the efficiency of butanol production.

## Methods

### Preparation of absorbents

#### Preparation of lignocellulosic carrier

*Pinus Radiata* was purchased from New Zealand. The wheat straw and corn stover were harvested from the suburb of Beijing, China. After air-dried, these three materials were chopped into 2.0-4.0 cm in length. Steam explosion pretreatment was carried out in a 7.5 L batch reactor (Weihai Automatic Control Reactor Ltd., China) as described in a previous work [30]. About 200 g prepared materials were soaked in 200 mL distilled water for 15 min, and then fed into the reactor. The pretreatment conditions for different materials were different, and it was operated at 1.8 MPa for 20 min for wood.

The steam-explosion pretreatment severity (log_10_*R*) can be calculated from the following equation [31]:

(1) $$R=t\;\exp\left(\frac{T-100}{14.75}\right),$$

where *R* is the reaction severity, *t* is the reaction time (min), and *T* is the reaction temperature (°C).

After steam explosion, the material was washed with 1 L of 80°C water and filtered by nylon cloth (200 mesh). Washed steam-exploded corn stover (SECS), wheat straw (SEWS) and steam-exploded wood (SEW) were dried separately at 60°C until constant weight.

For alkaline treatment, the SEWS, SECS or SEW was soaked in distilled water containing 1% NaOH (w/v) for 24 h at room temperature, and the solid substrate concentration was 10% (w/v). Solid residues were thoroughly washed with water until neutral pH, and then filtered through the nylon cloth (200 mesh). Alkali-treated SECS (SECSAT), SEWS (SEWSAT) and SEW (SEWAT) were dried in an oven at 60°C to constant weight and stored at room temperature.

Alkaline peroxide treated SECS (SECSAP) was obtained by soaked in distilled water containing 1% NaOH (w/v) and 4% H_2_O_2_ for 24 h at room temperature.

#### Preparation of hydrophobic carrier

Non-woven fabric was chosen as the hydrophobic substrate.

#### Preparation of hydrophilic carrier

Polyurethane foam was chosen as the hydrophilic substrate. The porosity was 96%, the pore size was 0.3-0.5 mm, and the density was 0.04 g/cm^3^. Polyurethane foam was cut into 5×5×5 mm cubes.

### Measurement of physical properties of absorbents

Specific gravity, bulk density, porosity and specific surface area were measured as described in former studies [32,33]. The pore size distribution was measured and analyzed by mercury porosimetry method using a mercury porosimeter (Micromeritics Autopore IV, Micromeritics, USA) [18].

### Microorganism and fermentation

*Clostridium acetobutylicum* ATCC 824 was maintained as a spore suspension in 6% (w/v) corn mash at 4°C. The pre-culture medium contained the following components per liter of distilled water: 30 g glucose, 4.3 g CH_3_COONH_4_, 1.768 g KH_2_PO_4_, 2.938 g K_2_HPO_4_, 10 mg p-aminobenzoic acid, 10 mg biotin and 1 mL mineral salts solution [18]. The initial pH of the medium was adjusted to 6.5 ± 0.2 with 1M NaOH or 1M H_2_SO_4_. The medium was sterilized at 115°C for 15 min. Cells were grown anaerobically at 37°C for 20–36 h without agitation before being transferred into the fermentation medium.

Batch fermentation experiments were carried out in a 100-mL serum bottle with a 60 mL working volume loaded with a certain amount of absorbent [11]. The fermentation medium was the same as the seed culture medium except the carbon sources. The pH of the medium was adjusted to 6.5 ± 0.2 before autoclaving at 115°C for 15 min. After cooled to room temperature, the fermentation media were inoculated with 6 mL inocula, and then infused with filtered oxygen-free nitrogen gas to maintain strict anaerobic conditions. Cultures were incubated at 37°C for 72 h without agitation. Samples were withdrawn at intervals for ABE, acids, and sugars analysis. The carrier was collected using a plastic 60-mL syringe, at the front end of which several holes of 1 mm diameter were punched. All experiments were carried out at least in duplicate.

### Analytical procedures for butanol fermentation

Glucose and xylose were determined by high-performance liquid chromatography (Agilent 1200 HPLC, Agilent Technologies, USA) with an Aminex HPX-87H column (300 mm × 7.8 mm, Bio-Rad Laboratories Inc.) and a refractive index detector. The mobile phase was 0.5 mM H_2_SO_4_ at a flow rate of 0.5 mL/min at 65°C. Sugars utilization was calculated as the percentage of utilized sugars (g) divided by added sugars (g).

ABE and acids (acetic and butyric acids) were measured using a gas chromatography (7890A, Agilent Technologies, USA) equipped with a flame ionization detector (FID) and an HP-Innowax capillary column (30 m × 0.32 mm). Oven temperature was held at 85°C for 4.5 min, programmed at increments of 20°C per min until 170°C, the final temperature was held for 2.5 min. Both injector and detector temperatures were set at 250°C. Nitrogen was employed as the carrier gas and isobutanol was applied as the internal standard. Productivity was calculated as the maximum ABE concentration achieved (g/L) divided by the fermentation time and was expressed as g/(L^.^h). Yield was calculated as the total amount of solvents divided by the utilized amount of sugar and was expressed as g/g.

The concentration of *C. acetobutylicum* in the fermentation broth was measured at 600 nm (OD600) using an ultraviolet–visible spectrophotometer (UV-1240, Shimadzu Co., Kyoto, Japan). The measured OD was correlated to dry weight using an established proportional constant [34].

### Measurement of absorptivity of SECSAT

The measurement of absorptivity of the SECSAT to ABE solvent was carried out by the adsorption isotherm and adsorption kinetics analysis. The adsorption isotherm was measured as follows: 2 g substrate was mixed with 30 mL prepared ABE solution in a 150 mL triangular flask and shaken at 37°C for 48 h for equilibrium experiments. At the end of the equilibrium period, the liquid phase was sampled using a syringe with several holes of 1 mm diameter in the front to remove solids. The ABE concentration of the solution was measured using a gas chromatography. The amount of absorbed ABE per unit mass of the substrate at adsorption equilibrium, *Q*_e_ (mg/g), was calculated by the mass balance equation in the liquid phase as shown in Equation (2).

(2) $$Qe=\frac{\left(C0-Ce\right)V}{W},$$

where *C*_0_, *C*_e_ (g/L) represents the concentration of ABE solvent in the aqueous phase at initial and equilibrium (after 48 h) conditions, *V* (mL) is the volume of aqueous solution, *W* (g) is the mass of absorbent.

The adsorption kinetics experiments were carried out in a thermostat air bath with agitation at 150 rpm and 37°C. The adsorption process was investigated at the initial concentrations of butanol, acetone and ethanol were 14.435 g/L, 7.694 g/L and 2.402 g/L, respectively. Samples were withdrawn at intervals for ABE analysis.

The morphology of SECSAT was imaged by a field emission scanning electron microscope (SEM, JSM-6700F, JEOL, Japan), and the morphology of bacterial adherence on SECSAT was studied using environmental scanning electron microscopy (ESEM, KYKY-1600, KYKY Technology Development Ltd., China).

## Abbreviations

ABE: Acetone-Butanol-Ethanol; AF: Absorbent fermentation; SSF: Solid-state fermentation; SmF: Submerged fermentation; PUF: Polyurethane foam; SECS: Steam-exploded corn stover; SEWS: Steam-exploded wheat straw; SEW: Steam-exploded wood; SECSAT: Alkali-treated SECS; SEWSAT: Alkali-treated SEWS; SEWAT: Alkali-treated SEW; SECSAP: Alkaline peroxide treated SECS; BET: Brunauer-Emmett-Teller; SEM: Scanning electron microscope.

## Competing interests

Both authors declare that they have no competing interests.

## Authors’ contributions

Qin He carried out the bacterial growth, fermentation, absorption, physical properties measurement, data analysis and drafted the manuscript. Hongzhang Chen conceived of the study, participated in its design and coordination and offered assitance to draft the manuscript. All the authors have read and approved the final manuscript.
